# Impact of Annealing and Strain on Magnetic and Magnetocaloric Properties of FeNiMnSiGe High-Entropy Alloy Nanoribbons Prepared by Magnetron Co-Sputtering

**DOI:** 10.3390/nano16140873

**Published:** 2026-07-16

**Authors:** Serhii Vorobiov, Iryna Pazukha, Oleksandr Pylypenko, Kostyantyn Tyschenko, Iurii Volk, Oleksii Hunbin, Maksym Lisnichuk, Daria Kondrakhova, Vladimír Tkáč, Erik Čižmár, Vladimír Komanický

**Affiliations:** 1 P.J. Šafárik University in Košice, Park Angelinum 9, 041 54 Košice, Slovakiavladimir.komanicky@upjs.sk (V.K.); 2 Sumy State University, Kharkivska 116, 40007 Sumy, Ukraine; 3 Institute of Materials Research of the Slovak Academy of Sciences, Watsonova 47, 040 01 Košice, Slovakia

**Keywords:** magnetron co-sputtering, high-entropy alloys, nanoribbon arrays, magnetic properties, magnetocaloric effect, strain, heat treatment

## Abstract

Non-equiatomic high-entropy alloys (HEAs) are promising candidates for low-dimensional magnetocaloric applications. In this work, Fe_25_Ni_21_Mn_24_Si_13_Ge_17_ HEA-based nanoribbon arrays with spacings of 1 and 2 µm, together with companion thin films, were fabricated under the same technological conditions by magnetron co-sputtering from five sources. The effects of heat treatment and longitudinal strain (0–2%) on the structural, magnetic, and magnetocaloric properties were studied using TEM, SAED, AFM, and SQUID magnetometry. TEM, SAED, and AFM confirmed an amorphous structure with a single cubic-type short-range order and thermal stability up to 700 K. The nanoribbons exhibited shape-induced magnetic anisotropy that vanished at a 2 µm spacing, where the ribbons became magnetically decoupled. Within the elastic regime, longitudinal strain acted via magnetoelastic coupling, preserving the in-plane isotropy while enhancing the magnetic response above 100 K. Annealing at 700 K drove short-range atomic reordering and relieved fabrication-induced strain, increasing the saturation magnetization severalfold and raising the maximum isothermal magnetic entropy change −Δ*S_M_* of the 1 µm nanoribbons from 1.25 to 1.35 JK^−1^kg^−1^ (at 140 K, Δ*H* = 50 kOe). The results demonstrate that strain engineering and thermal processing provide distinct, complementary routes for tuning the magnetic and magnetocaloric behavior of FeNiMnSiGe HEA nanoribbons.

## 1. Introduction

In developing prototypes of magnetic refrigerators, researchers are actively addressing key challenges that currently limit this technology’s potential. These challenges include: increasing the magnetocaloric effect of materials at low applied magnetic field values; reducing the heat exchange time between the material and the heat exchanger; minimizing unwanted losses due to magnetic hysteresis; enhancing the mechanical stability of magnetic materials; implementing intelligent thermal regulation and expanding the operating temperature range by creating cascade systems composed of multiple magnetocaloric materials [1]. To address these issues, the main strategies include tuning the chemical composition of magnetocaloric materials, employing multi-stimuli (temperature, pressure, and magnetic field), and reducing size [2,3].

Currently, most research is focused on the search for new magnetocaloric materials, the characterization of their properties, and the development and evaluation of potential magnetic refrigeration devices [2,4]. The overwhelming majority of these studies deal with bulk materials–arc-melted ingots, powders, or melt-spun ribbons tens of micrometers thick. In such macroscopic forms, the low surface-to-volume ratio limits the heat-exchange rate between the refrigerant and the working fluid, a key factor that restricts the operating frequency of a magnetic cooling cycle [1,3]. By contrast, low-dimensional structures: thin films, nanoparticles, and, in particular, lithographically patterned nanoribbon arrays, receive far less attention, even though their high surface-to-volume ratio, short thermal-diffusion length, and compatibility with micro- and on-chip cooling architectures make them attractive for miniaturized magnetocaloric devices [3]. Equally important, the low-dimensional geometry itself provides additional, independent handles for tuning the magnetic response that are unavailable in the bulk: shape-induced magnetic anisotropy set by the ribbon geometry and inter-ribbon spacing, and magnetoelastic coupling that can be controlled reversibly by external strain applied through a flexible substrate [5].

High-entropy alloys (HEAs) belong to a class of advanced multifunctional materials, which typically consist of five or more elements in approximately equal molar ratios (equiatomic HEAs) [1]. This composition promotes the formation of a homogeneous phase with high mixing entropy, stabilizing simple crystal structures such as face-centered cubic (fcc) or body-centered cubic (bcc) [6,7]. The functional properties of equiatomic HEAs are generally more limited than those of traditional materials [7,8]. Non-equiatomic alloys have recently gained popularity; in these alloys, the elements are present in different molar ratios, allowing for a more flexible approach to developing alloys with unique functional characteristics that equiatomic alloys cannot achieve [7,8]. By varying the concentration of individual components, it is possible to obtain not only solid solutions (as in equiatomic HEAs), but also intermetallics and ceramic compounds. This opens up a wide range of possibilities for optimizing functional characteristics, including catalytic, magnetic, electrical, and magnetothermal properties. Thin-film non-equiatomic HEAs could be an excellent alternative to traditional magnetocaloric materials. They possess exceptional mechanical properties and, combined with their cost-effectiveness (achieved by substituting expensive rare-earth elements with more accessible and common components), make them ideal candidates for practical use. Moreover, non-equiatomic HEAs have the potential to optimize their magnetocaloric properties by fine-tuning composition, thereby opening new opportunities for the development of efficient, economically viable magnetic refrigeration devices [5].

Classical magnetocaloric materials, which exhibit a significant magnetocaloric response, often contain rare-earth elements [9,10]. This raises concerns about their widespread practical application, given the high cost and limited availability of these resources. Rare-earth-free high-entropy alloys present an attractive alternative. In particular, non-equiatomic FeMnNiGeSi-type HEAs have been reported to exhibit a notable magnetocaloric response [11,12,13], approaching that of classical rare-earth compounds. It should be emphasized, however, that these results were obtained on bulk samples (arc-melted ingots or melt-spun micro-ribbons), which crystallize into an ordered single-phase hexagonal (hcp) structure and undergo a first-order magnetostructural transition responsible for their large, but sharp and hysteretic, entropy change [11,12,13]. The behavior of the same element composition in a genuinely low-dimensional form (nanoscale thin films and lithographically patterned nanoribbons) remains largely unexplored. Also, the search for the optimal composition, a pivotal aspect, is therefore how external factors (heat treatment, hydrostatic pressure, and mechanical deformation) reshape the magnetic and magnetocaloric properties of these low-dimensional HEAs.

To achieve optimal parameters for new HEAs capable of addressing the set tasks, it is proposed to use co-deposition magnetron sputtering with 5–6 sources. This approach offers a significant advantage over the classical method of magnetron sputtering deposition from a single sintered target, where a pre-prepared target with a specified concentration is used, due to its flexibility. In our case, there is no need to specially manufacture a target with a specific composition that cannot be easily changed if needed. During co-deposition magnetron sputtering from multiple sources, it is possible to flexibly adjust not only the stoichiometry of HEAs but also other parameters, such as substrate temperature, pressure, and atmospheric composition (by adding additional gases).

Most studies on magnetocaloric HEAs pursue a single optimization route, either compositional modulation, i.e., searching for the stoichiometry that maximizes the entropy change, or a single post-synthesis heat treatment [5,11,12,13]. Such single-knob strategies are effective for shifting the transition temperature or amplitude, but they cannot independently address the two distinct sources of magnetic disorder that dominate in sputter-deposited low-dimensional HEAs: the internal fabrication-induced stress on the one hand, and the frozen short-range atomic disorder of the as-deposited amorphous matrix on the other. Here, we adopt a different approach applied to the same co-sputtered material. Reversible elastic strain, transmitted through a flexible substrate, acts non-destructively through magnetoelastic coupling and probes the stress-sensitive part of the magnetic response, whereas thermal annealing acts irreversibly, driving short-range atomic reordering and relieving internal stress. Because the two stimuli operate through different physical mechanisms, they provide complementary and separable control over the magnetic and magnetocaloric behavior.

For these reasons, the present study deliberately targets a non-equiatomic, rare-earth-free FeNiMnSiGe composition in a low-dimensional form, while the thin-film and nanoribbon geometry is chosen for its fast heat exchange, its potential for device miniaturization, and the additional shape-anisotropy and strain-based tuning routes it enables. In this work, we investigated the effects of heat treatment and strain on the magnetic and magnetocaloric properties of FeNiMnSiGe HEA-based nanoribbons and thin films prepared by magnetron co-sputtering under identical conditions.

## 2. Materials and Methods

Fe_25_Ni_21_Mn_24_Si_13_Ge_17_ HEA-based nanoribbon arrays were obtained by combining electron-beam lithography with magnetron co-sputtering. Before lithography, the substrates were sequentially cleaned with acetone, isopropanol, and deionized water. To form a lithographic mask on the surface of (100) silicon substrates, positive resist, AR-P 6200.09 (CSAR 62, Allresist GmbH, Strausberg, Germany), was spin-coated with the following parameters: rotation speed 4000 rpm, acceleration 1000 rpm·s^−1^, application time 60 s. After applying the resist, the samples were subjected to a preliminary annealing at 160 °C for 60 s. The mask configuration was set using the DrawBeam software. For the electron-beam lithography, a Tescan VEGA scanning electron microscope (SEM) was used. The exposure field was 4 mm × 4 mm. To reduce the influence of beam aberrations and to ensure high quality of the formed elements, the exposure field was divided into smaller fragments with dimensions of 0.5 mm × 0.5 mm. During exposure, the following parameters were used: beam current (~1.5 nA), exposure step (pitch) 125 nm, exposure time 1 ms, and a standard irradiation dose of 200 μC/cm^2^. After exposure, the resist was placed in a developer AR 600-546.

To form nanoribbon arrays and thin films by magnetron sputtering, an ATC-Orion 8 Sputtering System (AJA International, Hingham, MA, USA), equipped with four DC magnetrons and one RF magnetron, was used. The base pressure in the vacuum chamber was 7 × 10^−8^ Torr. Deposition was carried out in an argon atmosphere. Deposition was carried out in an argon atmosphere (99.999%, Messer, Bratislava, Slovakia) at a gas flow rate of 18 sm^3^·min^−1^ and a constant base pressure of 3 mTorr.

In order to obtain the required non-equiatomic composition of Fe_25_Ni_21_Mn_24_Si_13_Ge_17_, a preliminary calibration of the power outputs of all five magnetrons was carried out. In the first stage, the required thickness of the deposited layer for each component was calculated to ensure the specified atomic concentration in the alloy, using the following equation:

(1) $$c_{i}=\frac{\rho_{i}d_{i}\mu_{i}^{-1}}{\sum_{i=1}^{n}\rho_{i}d_{i}\mu_{i}^{-1}}$$

where *c_i_* is atomic concentration of *i*-th component and *d_i_*, *μ_i_*, *ρ_i_* are the thickness, density and molar mass of the deposited material, respectively.

Once the required thicknesses had been determined, the power of each magnetron was selected based on a pre-established relationship between the deposition rate of the relevant material and magnetron power. This ensured that the calculated thickness of each component was achieved within the fixed deposition time.

After each deposition cycle, the chemical composition of the thin films was determined using energy-dispersive X-ray spectroscopy (EDS). The results obtained were used to adjust the power of the relevant magnetrons, after which a second deposition and EDS analysis were carried out. The iterative procedure was repeated until the required alloy composition Fe_25_Ni_21_Mn_24_Si_13_Ge_17_ was achieved.

Following the calibration, deposition was carried out using four DC magnetrons with targets Fe (142 W), Ni (78 W), Mn (72 W), and Ge (40 W), as well as one RF magnetron with a Si target (200 W). The deposition time was 160 s. During the deposition process, the substrate holder rotated continuously at 30 rpm about its axis, ensuring a uniform supply of material from all magnetrons and preventing compositional gradients from forming across the sample surface. Glass-carbon substrates (HTW Gmb, Thierhaupten, Germany) were used for the EDS analysis, thereby avoiding substrate signal and ensuring that the elemental composition of the alloy was determined correctly.

After deposition, the lift-off process was carried out by immersing the samples in AR 600-71 remover (Allresist GmbH) at room temperature (21 °C) and placing them in an ultrasonic bath for 90 s. Subsequently, the samples were rinsed with isopropanol and distilled water, and finally dried under an argon gas stream. As a result, nanoribbon arrays with the designed geometry and periodicity were successfully obtained.

**Figure 1:**
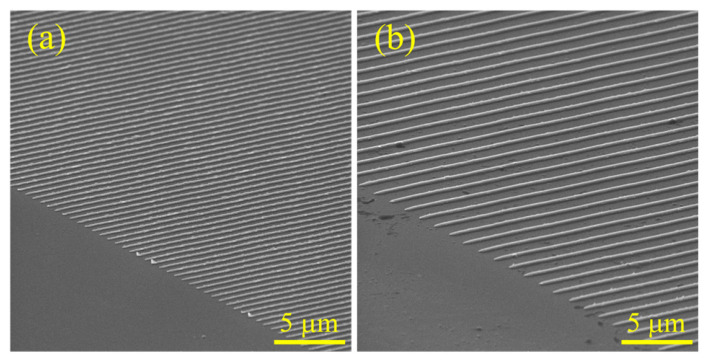
SEM micrographs of periodic linear nanostructures of Fe_25_Ni_21_Mn_24_Si_13_Ge_17_ HEA-based nanoribbon arrays with varying spacings: 1 µm (**a**) and 2 µm (**b**).

**Figure 2:**
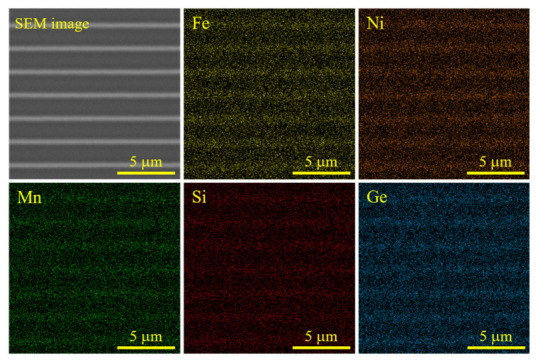
SEM micrographs and elemental mapping analysis of Fe_25_Ni_21_Mn_24_Si_13_Ge_17_ HEA-based nanoribbon arrays.

**Figure 3:**
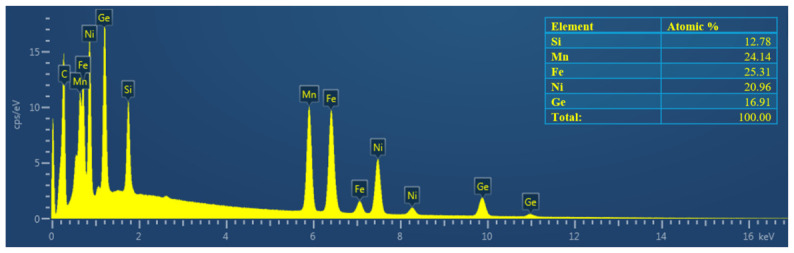
EDX analysis of Fe_25_Ni_21_Mn_24_Si_13_Ge_17_ HEA-based nanoribbon arrays.

Figure 1 shows a typical SEM image of a nanoribbon array after completion of all stages of nanofabrication. The dimensions of the nanoribbons were: length–500 µm, width–300 nm, height–35 nm. The homogenous distribution of constituent elements is presented in Figure 2. The presence of constituent elements is confirmed by EDX (Figure 3).

A transmission electron microscope (TEM), JEOL 2100F UHR (JEOL, Tokyo, Japan), operated at 200 kV with a Field Emission Gun (FEG), was used to investigate the microstructure of nanomagnets. Image characterization was performed in scanning/transmission modes using a bright-field detector. Phase identification was confirmed by Selected Area Electron Diffraction (SAED).

An atomic force microscope (AFM) Dimension Icon^®^ (Bruker, Billerica, MA, USA) was used to study the surface topography of the samples after deposition and heat treatment.

Magnetic measurements in zero-field-cooled (ZFC) and field-cooled (FC) modes were performed using a SQUID magnetometer (Quantum Design MPMS3, San Diego, CA, USA) in the temperature range 2–300 K under an applied magnetic field of 1 kOe. In the ZFC protocol, the sample was cooled to 2 K in zero field, after which the magnetic field was applied, and magnetization was measured upon warming to 300 K. Subsequently, the sample was cooled and measured again in the FC mode under the same applied field. Measurements were carried out with the magnetic field applied parallel and perpendicular to the nanoribbon axis.

To evaluate the magnetocaloric properties, isothermal magnetization curves were measured in magnetic fields up to 50 kOe over the temperature range 5–200 K. The isothermal magnetic entropy change ∆*S_M_* has been calculated using the Maxwell relation [14]:

(2) $$\Delta S_{M}=\left(T,\Delta B\right)=\int_{B_{i}}^{B_{f}}\frac{\partial M\left(T,B\right)}{\partial T}dB$$

where *B_i_* and *B_f_* represent the initial and final magnetic fields, respectively.

To investigate the strain effect on the magnetic properties of HEA nanoribbons, we have used a measurement system that enables automated, controlled experiments in an inert gas environment. For this purpose, Kapton substrates 60 mm long and 5 mm wide with pre-formed copper contacts were used. The distance between the contacts was 10 mm. HEA thin films were deposited through a shadow mask with a 15 mm × 1 mm aperture. As a result, the active region of the sample between the contacts was 10 mm long and 1 mm wide, and was used for subsequent electromechanical measurements.

The tested samples underwent X “load-unload” cycles over the strain ranges *ε_l_*_1_ = 0–1% and *ε_l_*_2_ = 0–2%. Real-time measurement of the corresponding changes in resistance is achieved using a digital multimeter. The gauge factor (GF) represents the strain sensitivity of the samples and is defined as the ratio of the relative change in electrical resistance to the applied mechanical strain:

(3) $$GF=\;\frac{∆R/R_{0}}{\varepsilon_{l}}$$

where ∆*R* is absolute change in resistance under applied longitudinal strain *ε_l_* = Δ*l*/*l_i_* (*l_i_* is the initial sample length), *R*_0_ is the initial resistance.

The differential gauge factor (*GF_dif_*) was calculated at an infinitely small strain interval ∆*ε_li_* at the relative change in resistance corresponding to this range.

To investigate the effect of heat treatment on the magnetic properties of HEA nanoribbons, the samples were annealed at 700 K in an Ar + N_2_ (2%) gas mixture in a furnace with continuous gas flow. The data collection system comprises a thermocouple sensor connected to a millivoltmeter and an ohmmeter connected to the test sample via a two-point measurement scheme. A built-in temperature-sensor/controller circuit regulates the furnace’s temperature and heating rate. The heating rate was maintained at 10 K/min.

## 3. Results and Discussion

### 3.1. TEM and AFM Investigations

**Figure 4:**
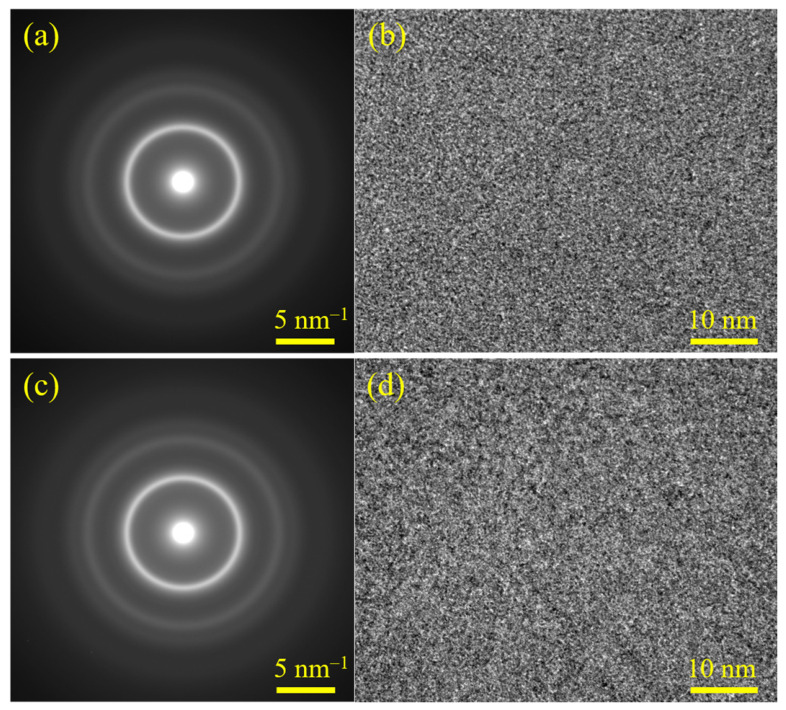
Electron diffraction patterns (**a**,**c**) and TEM images (**b**,**d**) of a 40 nm thick Fe_25_Ni_21_Mn_24_Si_13_Ge_17_ HEA-based thin film after deposition (**a**,**b**) and after annealing at 700 K (**c**,**d**).

Figure 4 shows electron diffraction patterns and TEM images of Fe_25_Ni_21_Mn_24_Si_13_Ge_17_ HEA after deposition at room temperature (RT) and annealing at 700 K. After deposition and annealing, the diffraction patterns exhibit only diffuse, broad concentric rings (Figure 4a,c), a signature of an amorphous state or an extremely fine, disordered ultra-nanocrystalline matrix where long-range atomic order is absent. This is confirmed by corresponding TEM imaging (Figure 4b,d). No distinct grain boundaries, large crystalline precipitates, or long-range lattice fringes are visible at this scale, confirming that the material maintains a highly disordered atomic configuration.

**Figure 5:**
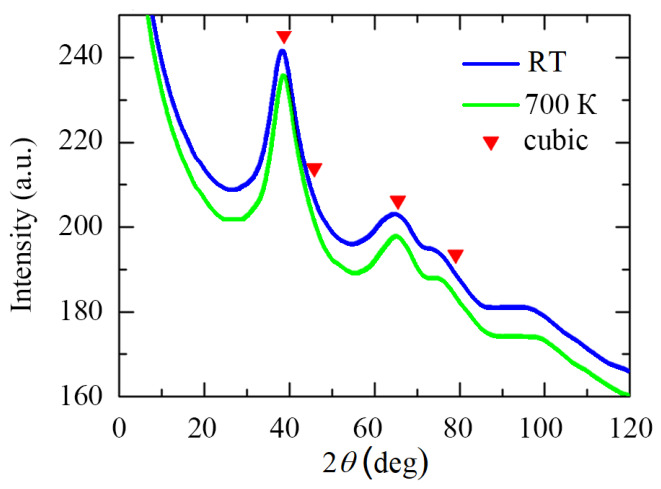
Electron diffraction profiles from a 40 nm thick Fe_25_Ni_21_Mn_24_Si_13_Ge_17_ HEA-based thin-film after deposition at room temperature (RT) and after annealing at 700 K.

To provide deeper insight into the structure of the HEA samples, the electron diffraction patterns were processed using specialized software and presented in the form of electron diffraction profiles (Figure 5). The diffraction peaks marked with red inverted triangles in Figure 5 correspond to a cubic crystal structure with 2*θ* = 38.5°, 45.0°, 65.0°, and 78°. The diffraction spectra at RT and 700 K are almost identical in shape, peak positions, and widths. This demonstrates that the Fe_25_Ni_21_Mn_24_Si_13_Ge_17_ HEA exhibits thermal stability, maintaining its single-phase cubic structure without phase decomposition, crystallization of the amorphous matrix, or precipitation of secondary intermetallic phases up to 700 K.

**Figure 6:**
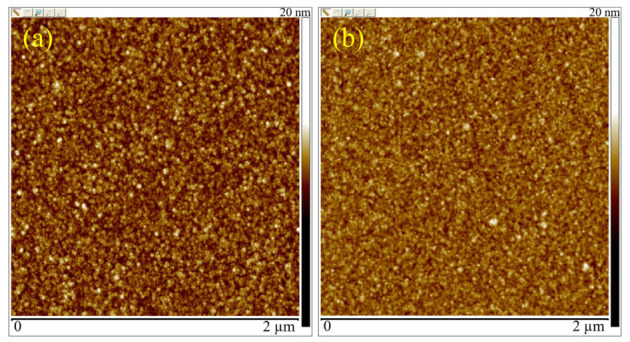
AFM of 40 nm thick Fe_25_Ni_21_Mn_24_Si_13_Ge_17_ HEA-based thin film after deposition (**a**) and after annealing at 700 K (**b**).

To investigate the surface morphology of Fe_25_Ni_21_Mn_24_Si_13_Ge_17_ HEA-based thin films and their evolution during annealing, atomic force microscopy analysis (Figure 6) was performed. As shown in Figure 6a, the surface exhibits a highly homogeneous nanostructured topography with a uniform spatial distribution. The morphology is characterized by densely packed, spherical nanometer-sized grains. Statistical evaluation of the height profile yields a root-mean-square roughness (*R*_q_) of 0.794 nm and an average roughness (*R*_a_) of 0.630 nm, indicating a high degree of surface planarity. Figure 6b illustrates the morphological modification resulting from thermal annealing at 700 K.

A subtle change in grain arrangement can be seen upon close inspection. This is shown by a slight tendency for individual clusters of many grains to coalesce in one place. However, the fundamental ultra-nanocrystalline or amorphous matrix is fully preserved. The integral roughness values (*R_q_* = 0.79 nm and *R_a_* = 0.63 nm) remain virtually unchanged within experimental error. This indicates excellent thermal stability of the nanostructure and the absence of severe recrystallization or grain growth at 700 K. These results are consistent with TEM analysis.

### 3.2. Magnetic Properties

**Figure 7:**
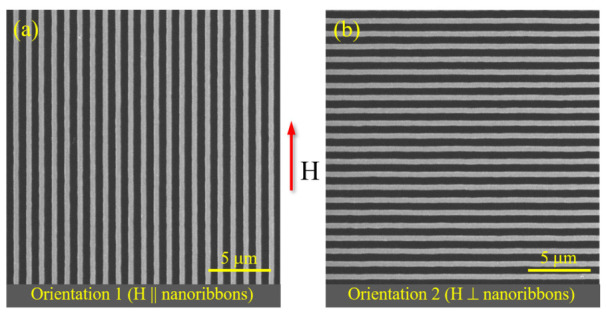
SEM images of the nanoribbon arrays under different geometric configurations relative to the external magnetic field (H, indicated by the red arrow): Orientation 1, where the magnetic field vector is parallel to the long axis of the ribbons (**a**); Orientation 2, where the magnetic field vector is perpendicular to the long axis of the nanoribbons (**b**).

The magnetic properties and magnetization dynamics were investigated under two distinct configurations relative to the external magnetic field (H), as shown in Figure 7. In Orientation 1 (Figure 7a), the long axis of the nanoribbon arrays is aligned vertically, running parallel to the direction of the applied magnetic field vector (H ‖ nanoribbons). This configuration is typically used to probe the easy magnetization axis driven by shape anisotropy. In Orientation 2 (Figure 7b), the array is rotated by 90°, positioning the nanoribbon arrays horizontally so that their longitudinal axis is perpendicular to the applied magnetic field vector (H ⊥ nanoribbons). This setup allows evaluation of the hard-axis behavior and quantification of demagnetizing factors across the nanoribbon’s width.

The high geometric consistency between the two orientations, as confirmed by the 5 µm scale bars, ensures that any observed differences in magnetic parameters (such as coercivity, remanence ratio, or saturation fields) are intrinsic to the directional magnetic anisotropy of the nanoribbons rather than structural artifacts or variations in pattern density.

**Figure 8:**
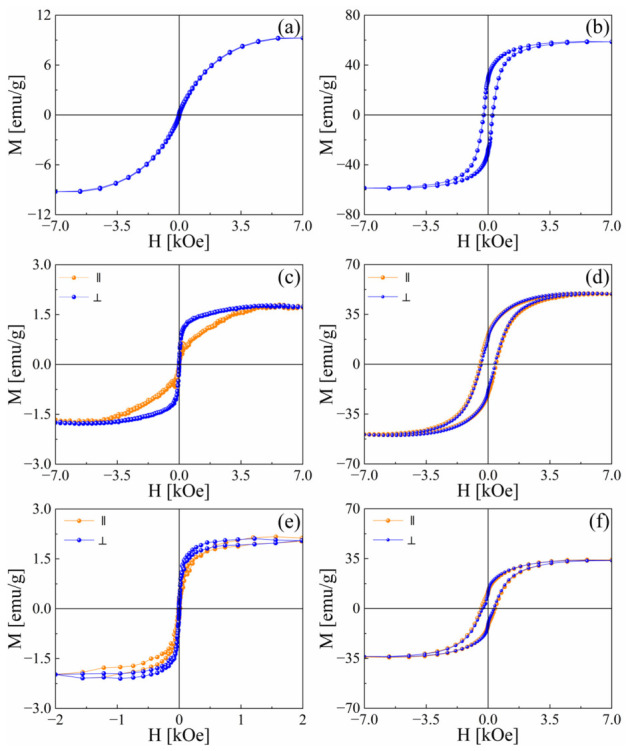
Hysteresis loops of Fe_25_Ni_21_Mn_24_Si_13_Ge_17_ HEA-based thin film (**a**,**b**) and nanoribbons with spacings of 1 µm (**c**,**d**) and 2 µm (**e**,**f**) in the initial state measured at 300 K (**a**,**c**,**e**) and 5 K (**b**,**d**,**f**).

Figure 8 shows the *M*(H) hysteresis loops measured at 300 K and 5 K in an external field for Fe_25_Ni_21_Mn_24_Si_13_Ge_17_ HEA-based thin films and nanoribbon arrays with spacings of 1 µm and 2 µm in the initial state. The experimental curves reveal a pronounced temperature-dependent evolution of both the saturation magnetization and the hysteretic response. At room temperature, the thin film exhibits a narrow hysteresis loop with negligible coercivity and low remanent magnetization, which is characteristic of a soft ferromagnetic state. The magnetic moment approaches saturation relatively slowly, reaching a maximum saturation magnetization *M*_s_ of 9 emu/g at high magnetic fields. Upon cooling the sample to 5 K, a significant enhancement in the magnetic properties is observed. The saturation magnetic moment increases drastically, reaching 60 emu/g. Furthermore, the loop broadens noticeably, exhibiting a distinct open structure with a clear coercive *H*_c_ of 2.4 kOe and a higher remanence ratio, *M*_r_/*M*_s_. This significant increase in both the saturation magnetization and coercivity at low temperatures indicates a strong reduction in thermal fluctuations (thermal activation effects) and the subsequent freezing of magnetic moments or suppression of domain wall motion at 5 K, thereby strengthening the effective magnetic anisotropy of the thin-film system [15].

The character of *M*(*H*) curves for Fe_25_Ni_21_Mn_24_Si_13_Ge_17_ HEA-based nanoribbon arrays differs from that of thin films. Such structures exhibit magnetic anisotropy, as evidenced by hysteresis loops measured at 300 K (Figure 8c,e) [16]. As presented in Figure 8c,e, the magnetization process depends on the orientation of the applied magnetic field to the axis of the nanoribbons. In perpendicular orientation, the more rapid remagnetization in the low-field region and higher residual magnetization are observed. At the same time, in parallel orientation, magnetization occurs more gradually. Such behavior agrees with magnetic anisotropy arising from geometric constraints and the domain structure of the nanoribbon arrays.

The value of *M*_s_ decreases to 1.75 emu/g and 2.1 emu/g for nanoribbon arrays with spacings of 1 µm and 2 µm, respectively. At 5 K, the saturation magnetization *M*_s_ also decreases compared with data for thin films, reaching values of 50 emu/g and 35 emu/g for nanoribbon arrays with spacings of 1 µm and 2 µm, respectively. This significant decrease in magnetization indicates increased surface spin frustration and domain pinning along the edges of the nanoribbons. In addition, at 5 K, the nanoribbon arrays with a spacing of 1 µm (Figure 8d) show a slight anisotropy in approach to saturation. However, for nanoribbon arrays with a spacing of 2 µm (Figure 8f), curves at the parallel and perpendicular orientations perfectly overlap. This reveals that increasing the spatial separation to 2 µm decouples the nanoribbons, causing the array to exhibit isotropic in-plane behavior at low temperatures.

**Figure 9:**
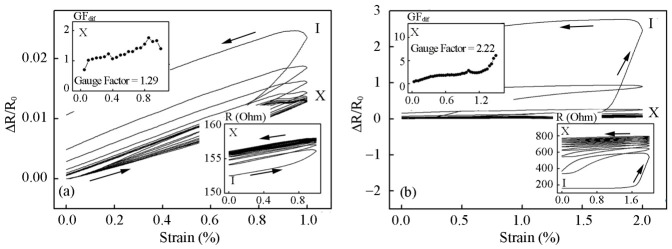
Δ*R*/*R*, GF, and *R* as a function of the longitudinal strain *ε_l_* for 40 nm thick Fe_25_N_i21_Mn_24_Si_13_Ge_17_ HEA-based thin film (**a**,**b**) within X strain cycles. Strain rage, %: (0–1) (**a**) and (0–2) (**b**).

Figure 9 illustrates the response-strain curve of the Fe_25_Ni_21_Mn_24_Si_13_Ge_17_ HEA thin film within the strain range *ε_l_*_1_ = 0–1% and *ε_l_*_2_ = 0–2%. For strain range *ε_l_*_1_ = 0–1% (Figure 9a), the Δ*R*/*R*(*ε_l_*) dependences are characterized by a significant difference in the I-st strain cycle from the subsequent cycles. The reason for this behavior is a stabilization process involving microplastic strain, redistribution, and the movement of defects in the crystalline structure and foreign atoms [17,18,19]. Starting with the second strain cycle, a tendency toward stabilization of the Δ*R*/*R*(*ε_l_*) curves is observed. For strain range *ε_l_*_1_ = 0–1%, a nearly linear increase in resistance with increasing strain is observed at the X-th strain cycle. So, elastic deformation occurs up to *ε_l_* = 1%. For this reason, the strain range was increased to 2% (Figure 9b). For this strain range, another characteristic feature is that the first strain cycle differs from the subsequent ones. In subsequent cycles, a stabilization of the strain-stress relationships is also observed. The results demonstrate gauge factors of 1.29 and 2.22 for strain ranges *ε_l_*_1_ = 0–1% and *ε_l_*_2_ = 0–2%, respectively. The electrical resistance behavior under strain confirms that the co-sputtered HEA thin film possesses desirable properties, including compositional homogeneity, surface uniformity, and a dense structure. These characteristics enable efficient and stable strain transfer within the composite structure. These properties guarantee optimal and consistent strain transfer within the HEA thin films. As elastic deformation occurs up 2%, we decided to analyze the effect of strain on magnetic properties over the range *ε_l_*_2_ = 0–2%. Further increasing the strain value is inadvisable, as it may cause the nanoribbons to crack.

**Figure 10:**
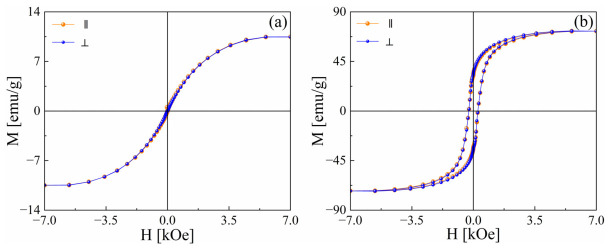
Hysteresis loops of 40 nm thick Fe_25_Ni_21_Mn_24_Si_13_Ge_17_ HEA-based thin film after stress, measured at 300 K (**a**) and 5 K (**b**).

The magnetic behavior of the HEA-based thin film after strain within the range 0–2% is presented in Figure 10. As shown in Figure 10, after stress, the thin film retains its strong temperature-dependent magnetization, though a minor decrease in the *M*_s_ to 56 emu/g at 5 K is observed compared with the samples before stress (Figure 8a,b). Furthermore, the overlap of the loops obtained from two different orientations (Figure 10) demonstrates that the stress does not break the in-plane magnetic isotropy of the thin film structure.

**Figure 11:**
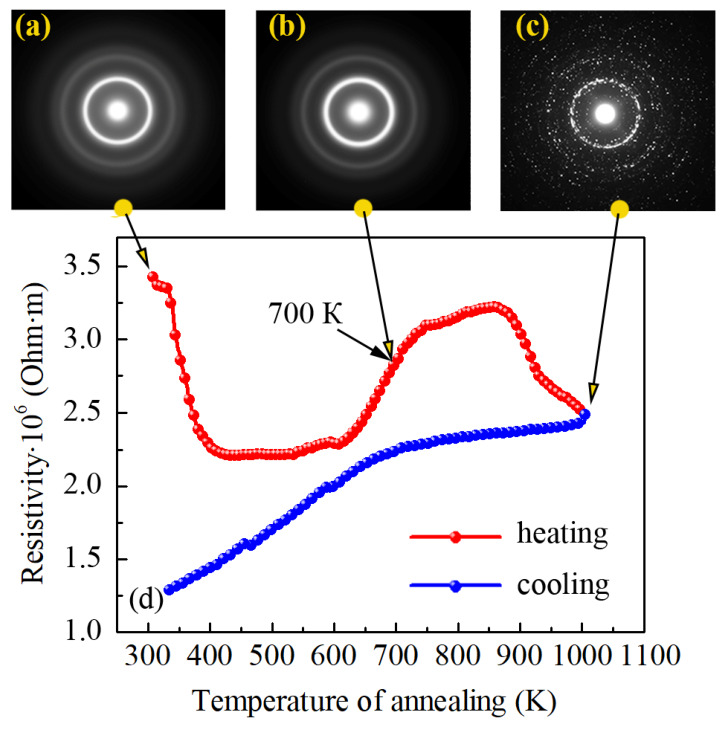
Electron diffraction patterns (**a**–**c**) and temperature dependence of resistivity during the cycle of “heating–cooling” within the temperature range 300–1000 K (**d**) for a 100 nm thick Fe_25_Ni_21_Mn_24_Si_13_Ge_17_ HEA-based thin film.

Resistivity is one of the base parameters affecting the magnetic, magnetotransport, etc., properties of thin film materials [20,21] and high-entropy alloys in particular [22]. Moreover, thermal stability remains an important research topic. So, a comprehensive investigation of the temperature dependence of resistance and changes in crystal structure under high-temperature annealing was conducted. The results are shown in Figure 11. The temperature dependence of the resistivity during heating (red line) shows a complex, non-monotonic behavior. Initially, the resistivity decreases sharply upon heating up to approximately 425 K, after which it stabilizes into a plateau. Beyond 625 K, resistivity increases significantly, reaching a local maximum near 855 K before dropping again, reaching a minimum at 1000 K, the maximum annealing temperature. In contrast, the subsequent cooling process (blue line) exhibits a monotonic, metallic-like linear decrease in resistivity down to room temperature, indicating an irreversible structural transformation. Therefore, the processes of electrical transfer in HEA thin films are driven by scattering of conduction electrons at grain boundaries, impurities, and phonons, as in electrically continuous metal layers [23].

To correlate the electron transport behavior with structural changes, the Selected Area Electron Diffraction patterns were studied at specific temperatures: after deposition, after annealing at a temperature of 700 K that corresponds to the region where resistivity begins to grow rapidly, and after annealing at a temperature of 1000 K that corresponds to the maximum of the annealing temperature (Figure 11a–c). Data for the 100-nm-thick HEA-based thin film correlate with those presented above. Before annealing, the SAED pattern exhibits broad, diffuse halo rings characteristic of a completely amorphous or highly disordered crystal structure (Figure 11a). At an annealing temperature of 700 K (Figure 11b), the diffraction rings remain diffuse, indicating that the amorphous matrix is still predominant or shifting into a precursor state. At the same time, upon reaching the maximum annealing temperature (Figure 11c), the diffuse halos completely transform into a series of sharp, dotted diffraction rings. This transition provides direct evidence of crystallization, confirming that the high-temperature thermal heat induces the formation of a polycrystalline phase.

Hence, based on the combined electrical resistivity and electron diffraction analysis, an annealing temperature of 700 K was chosen for further magnetic characterization. Investigating magnetic properties at this temperature allows for the study of a “metastable” or “pre-crystallization” state. This helps determine how structural relaxation, short-range ordering, or the very early nucleation of nanocrystals within the amorphous matrix affects magnetic behavior before the full-scale crystallization observed at 1000 K takes over. By selecting 700 K, we can decouple the effects of early-stage atomic rearrangement from the microstructural effects associated with large crystalline grains and dense grain boundaries.

**Figure 12:**
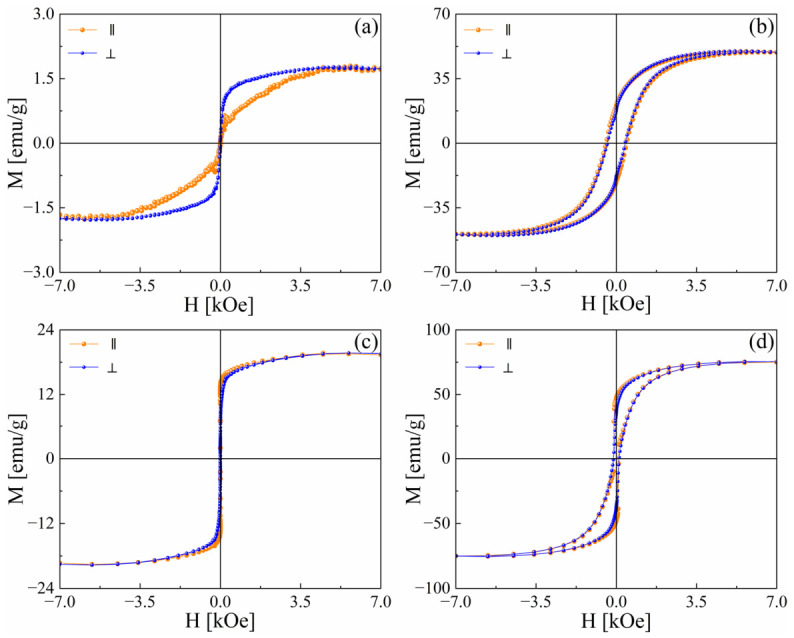
Hysteresis loops of Fe_25_Ni_21_Mn_24_Si_13_Ge_17_ HEA-based nanoribbons with a spacing of 1 µm before (**a**,**b**) and after annealing at 700 K (**c**,**d**), measured at 300 K (**a**,**c**) and 5 K (**b**,**d**).

Figure 12 shows the magnetic field-dependent magnetization loops for HEA-based nanoribbons with a spacing of 1 μm, measured at 300 K and 5 K before and after annealing at 700 K. Annealing the sample at 700 K leads to a significant change in the *M*_s_ value. At room temperature, *M*_s_ increases to approximately 19.5 emu/g, accompanied by the complete collapse of directional anisotropy as the parallel and perpendicular loops converge (Figure 12c). Similarly, at 5 K, heat treatment increases the maximum magnetization capability from approximately 50 emu/g in the initial state to approximately 75 emu/g after annealing (Figure 12b,d). These developments demonstrate that annealing at 700 K is a vital structural threshold for the HEA system, driving short-range atomic reordering and relieving fabrication-induced mechanical strain. This effectively lowers internal magnetic pinning barriers, establishes isotropic domain-rotation pathways, and significantly increases the total ferromagnetic volume fraction within the nanoribbons.

**Figure 13:**
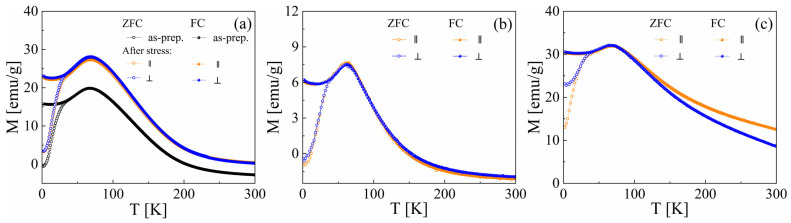
ZFC-FC magnetization curves measured in a 1 kOe measuring and cooling field applied parallel and perpendicular to the Fe_25_Ni_21_Mn_24_Si_13_Ge_17_ HEA-based thin film before and after strain (**a**) and nanoribbon arrays with a spacing of 1 µm before (**b**) and after annealing at 700 K (**c**).

Figure 13 shows the temperature dependence of the ZFC-FC magnetization for the Fe_25_Ni_21_Mn_24_Si_13_Ge_17_ HEA-based thin film before and after stress (Figure 13a), and for the nanoribbon arrays before and after annealing at 700 K (Figure 13b,c), measured in a field of 1 kOe.

For thin films, the ZFC-FC curves do not indicate direct evidence of the magnetic transition from the ferromagnetic to the paramagnetic state. Only a broad maximum, similar to that observed in superparamagnetic systems, was observed, with a well-defined peak at *T* ≈ 70 K, determined from the position of the maximum in the temperature dependence of the ZFC magnetization. The application of longitudinal stress does not alter the peak position, which remains at 70 K. This indicates that the thermal energy barrier underlying the main blocking transition in the magnetic clusters is unaffected by the strain. At this, it should be noted that, within the temperature range *T* > 100 K, a distinct modification in the slope of the ZFC-FC curves, with significantly higher magnetic moment up to room temperature, is observed after sample stress. This altered temperature decay can be explained by the emergence of magnetoelastic anisotropy [24] induced by residual internal stresses, which increases the energy barriers against thermal fluctuations and stabilizes the magnetic order at higher temperatures.

The ZFC-FC dependencies in the initial state do not depend on the orientation of the magnetic field relative to the axis of the nanoribbons (Figure 13b). The temperature value that corresponds to the peak on the ZFC-FC magnetization curve is 62 K. A pronounced magnetic anisotropy is visible across the entire temperature scale. However, subjecting the nanoribbons to thermal annealing at 700 K (Figure 13c) induces transformations in the magnetic structure. First, the peak specific magnetization increases more than fourfold, growing from 7.5 emu/g in the initial state to 32 emu/g after annealing, indicating a major expansion of the net ferromagnetic volume fraction. Second, while the temperature region *T* > 100 K is completely isotropic in the nanoribbons before annealing (Figure 13b), the annealed sample (Figure 13c) exhibits a prominent magnetic anisotropy up to room temperature, with the parallel orientation yielding significantly higher magnetization values than the perpendicular orientation. Additionally, the ZFC curves after annealing retain an elevated baseline magnetization at the lowest temperature limit (*T* → 0 K). Together, these findings demonstrate that annealing at 700 K promotes short-range atomic reordering and dissipates internal fabrication strains, thereby reducing local domain-pinning centers, enhancing collective exchange interactions, and establishing a robust in-plane magnetic easy axis along the nanoribbon length.

### 3.3. Magnetocaloric Properties

**Figure 14:**
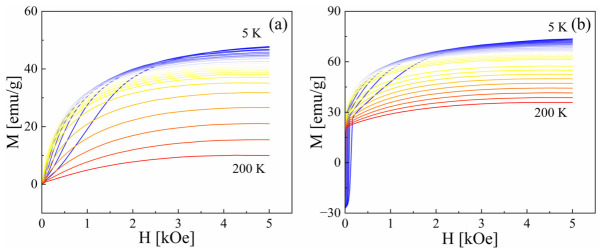
Magnetization as a function of applied magnetic field applied parallel to the Fe_25_Ni_21_Mn_24_Si_13_Ge_17_ HEA-based nanoribbon arrays with spacings of 1 µm under various temperatures before (**a**) and after (**b**) annealing at 700 K.

Figure 14 shows the isothermal magnetization and demagnetization curves for the Fe_25_Ni_21_Mn_24_Si_13_Ge_17_ HEA-based nanoribbons with a spacing of 1 µm over the applied magnetic field range of 0–5 kOe. Both states exhibit a typical soft-magnetic behavior, with magnetization gradually decreasing as temperature increases due to thermal fluctuations. However, a comparative analysis of *M*(H) curves for as-deposited (Figure 14a) and annealed at 700 K (Figure 14b) samples reveals a substantial enhancement in magnetic performance upon annealing. Specifically, the maximum magnetic moment at 5 K increases from 48 emu/g to 74 emu/g after annealing. Furthermore, the annealed sample exhibits a significantly steeper initial slope at lower fields (*H* < 1 kOe), pointing to enhanced magnetic susceptibility. These results indicate that annealing at 700 K promotes atomic reordering and structural relaxation within the HEA nanoribbon, effectively reducing structural pinning centers and optimizing the collective ferromagnetic alignment.

**Figure 15:**
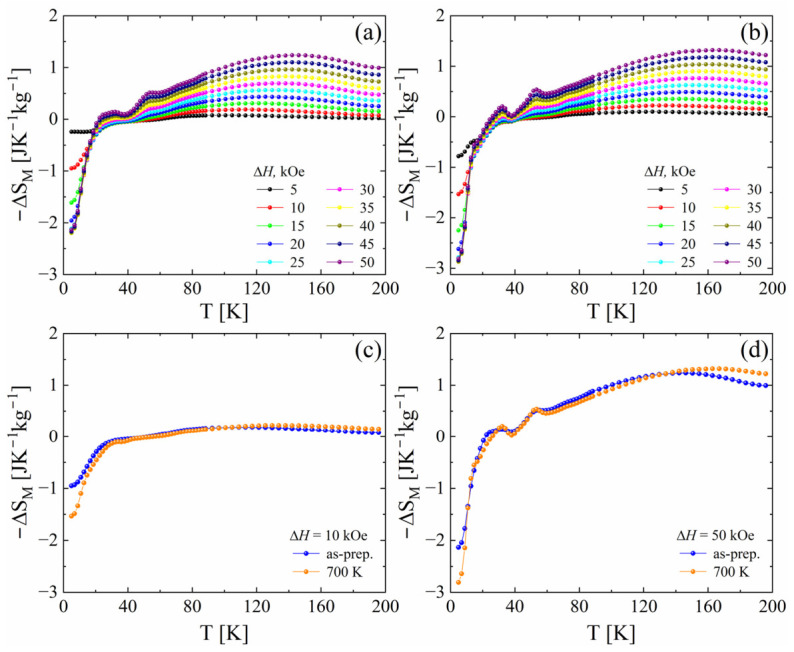
Dependence of the change in isothermal entropy (−Δ*S_M_*) in Fe_25_Ni_21_Mn_24_Si_13_Ge_17_ HEA-based nanoribbon arrays with spacings of 1 µm before (**a**) and after annealing at 700 K (**b**) on temperature under various magnetic fields applied parallel to the nanoribbon axis, and comparison of dependences −Δ*S_M_*(*T*) before and after annealing at Δ*H* = 10 kOe (**c**) and 50 kOe (**d**).

The magnetocaloric properties of the Fe_25_Ni_21_Mn_24_Si_13_Ge_17_ HEA-based nanoribbons with a spacing of 1 µm were evaluated by extracting the temperature dependence of the isothermal magnetic entropy change (−Δ*S_M_*(*T*)), as shown in Figure 15. The curves exhibit coexistence of an inverse magnetocaloric effect below 20 K and a broad conventional magnetocaloric peak spanning 40–200 K. The maximum value of the isothermal entropy change −∆*S_M_* = 1.25 JK^−1^kg^−1^ for parallel orientation was observed at 140 K and Δ*H* = 50 kOe. Heat treatment at 700 K increases the maximum value of −Δ*S_M_* to approximately 1.35 JK^−1^kg^−1^. This enhancement in the magnetic entropy change is highly consistent with the improved magnetization dynamics and structural homogenization induced by the 700 K annealing, which minimizes structural pinning defects and optimizes the spin-entropy variation.

It should be noted that the amplitude value of isothermal entropy, which characterizes HEAs, which do not contain rare-earth elements in their composition [11,12,13], is much higher than that obtained in our work. Namely, in FeMnNiGeSi HEAs, the values of isothermal entropy change as large as 13 JK^−1^kg^−1^ (for 25 kOe) [11], 7.3 JK^−1^kg^−1^ (for 25 kOe) [12], in MnNiSiFeCoGe HEAs, −16 JK^−1^kg^−1^ (for 20 kOe) are achieved. According to Refs. [11,12,13], all samples, unlike ours, exhibit a single-phase hcp structure at room temperature after preparation. In our view, this is one of the reasons why our results differ from those published. In this regard, the next stage of our research will involve determining the process conditions under which samples with an ordered crystal structure can be produced via magnetron co-sputtering. In combination with strain engineering and thermal processing, this will allow tuning the magnetic and magnetocaloric behavior of rare-earth-free FeNiMnSiGe HEA nanoribbons.

## 4. Conclusions

Fe_25_Ni_21_Mn_24_Si_13_Ge_17_ HEA-based nanoribbon arrays and thin films were fabricated under identical technological conditions by five-source magnetron co-sputtering. TEM, SAED, and AFM showed that the as-deposited material has an amorphous structure with a single cubic-type short-range order and a smooth surface (*R*_q_ = 0.79 nm, *R*_a_ = 0.63 nm). This structure remains thermally stable up to 700 K, without phase decomposition, recrystallization, or grain growth, whereas combined resistivity and electron-diffraction analysis revealed an irreversible transition to a polycrystalline phase only near 1000 K. Accordingly, 700 K was selected as a metastable, pre-crystallization state for the magnetic study.

The magnetic response is strongly temperature- and geometry-dependent. The thin film behaves as a soft ferromagnet, with a saturation magnetization *M*_s_ of 9 emu/g at 300 K, increasing to 60 emu/g at 5 K, and a coercivity *H*_c_ of 2.4 kOe at 5 K. The nanoribbon arrays exhibit shape-induced magnetic anisotropy that vanishes at a 2 µm spacing, where the ribbons become magnetically decoupled, and the array becomes isotropic in-plane. Within the elastic regime (gauge factors of 1.29 and 2.22 for the 0–1% and 0–2% strain ranges), longitudinal strain acts predominantly through magnetoelastic coupling. It preserves the peak in the ZFC-FC magnetization curves and in-plane isotropy but introduces magnetoelastic anisotropy that stabilizes the magnetic order above 100 K. In contrast, annealing at 700 K triggers short-range atomic reordering and relieves fabrication-induced strain, increasing the peak ZFC magnetization of the nanoribbons more than fourfold (from 7.5 to 32 emu/g) and establishing a robust in-plane easy axis along the ribbon length. Overall, strain engineering and thermal processing thus provide distinct and complementary routes for tuning the magnetic behavior of FeNiMnSiGe HEA.

These structural improvements directly enhance the magnetocaloric performance of the 1 µm nanoribbons: the maximum magnetic moment at 5 K rises from 48 to 74 emu/g, and the maximum isothermal magnetic entropy change −Δ*S_M_* increases from approximately 1.25 to 1.35 JK^−1^kg^−1^ (at 140 K, Δ*H* = 50 kOe). The −Δ*S_M_*(*T*) curves reveal an inverse magnetocaloric effect below 20 K and a broad conventional peak between 40 and 200 K.

The low-dimensional nanoribbon geometry represents one of the main practical advantages of the present materials. Owing to their high surface-to-volume ratio and short thermal diffusion length, such nanostructures are expected to facilitate rapid heat exchange with the working fluid and therefore may support higher operating frequencies than bulk counterparts. Their lithographically defined periodic geometry is compatible with standard microfabrication processes, making them attractive for on-chip cooling systems, MEMS devices, and microfluidic heat exchangers. Furthermore, the broad and weakly hysteretic −Δ*S_M_* plateau (≈40–200 K) is potentially advantageous for Ericsson-type regenerative cycles and cascade magnetocaloric refrigerators, where a wide entropy-change profile is preferred over a narrow first-order peak. Combined with the rare-earth-free composition, these characteristics suggest a promising route toward compact, low-cost, and mechanically robust magnetocaloric micro-refrigeration technologies.

Beyond magnetocaloric applications, the co-sputtered material demonstrates potential as a flexible functional thin-film sensing element. Deposited on Kapton substrates, the films exhibit a stable and reproducible strain response with conventional metallic gauge factors. Combined with the amorphous structure that remains thermally stable up to 700 K and the excellent mechanical properties generally associated with high-entropy alloys, the present films appear promising for strain sensing in flexible or elevated-temperature applications.

At the same time, the principal limitation of the present rare-earth-free HEA nanoribbons is the modest amplitude of the entropy change (−Δ*S_M_* ≈ 1.35 JK^−1^kg^−1^), roughly an order of magnitude below that of bulk FeMnNiGeSi HEAs [11,12,13]. This behavior can be attributed to the amorphous, second-order-like character of the as-deposited state and the reduced effective magnetic volume resulting from the lithographically patterned nanoribbon geometry. Several optimization strategies follow directly from these findings: (i) tuning the deposition and annealing protocol to stabilize the ordered crystal structure, and hence the first-order magnetostructural transition, while preserving the low-dimensional form; (ii) shifting the operating temperature toward room temperature through further compositional adjustment enabled by the flexible 5-source co-sputtering; (iii) increasing the active magnetic volume by optimizing ribbon thickness, width, and packing density; and (iv) exploiting substrate clamping and controlled residual strain as a permanent, built-in magnetoelastic bias. Realizing an ordered crystalline phase directly by magnetron co-sputtering is the immediate next step of our work, as it is expected to combine the large entropy change of the bulk counterparts with the geometric, thermal, and mechanical advantages of the nanoribbon architecture.

## Data Availability

The original contributions presented in this study are included in the article. Further inquiries can be directed to the corresponding author.
